# Post-Surgical Abdominal Myonecrosis: The Unusual Role of Candida albicans

**DOI:** 10.7759/cureus.84273

**Published:** 2025-05-17

**Authors:** Brian Musch, Rachel A Daley, Alyssa McMandon, Saptarshi Biswas

**Affiliations:** 1 Surgery, Grand Strand Medical Center, Myrtle Beach, USA; 2 Medicine, Edward Via College of Osteopathic Medicine, Spartanburg, USA

**Keywords:** candida albicans, elective surgery complication, emergent general surgery, giant ventral hernia, necrotizing myonecrosis

## Abstract

Necrotizing myonecrosis is a life-threatening infection of the skeletal muscle and soft tissues, predominantly caused by bacteria such as *Staphylococcus aureus* and *Group A Streptococcus*. In rare cases, fungal organisms, particularly *Candida albicans*, have been identified as the sole causative agent in these infections. We present a rare case of a 77-year-old female patient who underwent an elective hysterectomy that was complicated by an iatrogenic injury to her small bowel, which was missed in the preliminary surgery. Postoperatively, she developed signs of peritonitis and greenish-brown drainage from her incision site. Exploratory laparotomy confirmed small bowel perforation. Subsequently, she developed abdominal sepsis and necrotizing myonecrosis involving the anterior abdominal wall musculature. Empiric treatment with broad-spectrum antibiotics was initiated. Intraoperative cultures revealed isolated *Candida albicans*, prompting a shift in management to antifungal therapy and multiple surgical debridements of the anterior musculature.

## Introduction

Necrotizing myonecrosis occurs when an infection or trauma causes the death of the skeletal muscle and surrounding tissues, often due to inadequate blood supply to the affected area. It is typically characterized by a triad of swelling, erythema, and disproportionately severe pain. Disproportionate pain is a serious sign that warrants urgent surgical referral and intervention [1]. Systemic manifestations of shock can include fever, hypotension, and tachycardia [2]. Left untreated, necrotizing myonecrosis can be potentially life-threatening. Approximately one-quarter to one-half of patients with necrotizing infections develop septic shock and/or require mechanical ventilation, while one-third develop acute kidney injury [3]. 

Early recognition and aggressive surgical debridement with excision of necrotic tissue are crucial to reducing mortality and preventing further complications [4]. Cultures obtained from the interface of necrotic and healthy tissue during the initial debridement, alongside blood cultures, are paramount to identifying the causative organisms and guiding medical management [5]. Necrotizing myonecrosis is often polymicrobial, frequently involving bacteria such as *Staphylococcus aureus, Streptococcus pyogenes*, and *Clostridium perfringens*. In rare cases, fungal organisms, particularly *Candida albicans,* have been isolated as the causative agent, necessitating aggressive management. 

We describe a rare case of an elderly female in relatively good health for her age who underwent total abdominal hysterectomy and bilateral salpingo-oophorectomy. Postoperatively, she developed necrotizing myonecrosis, with cultures revealing isolated *Candida albicans*, necessitating extensive surgical debridements. This case underscores the importance of considering fungal pathogens as a probable etiology for necrotizing myonecrosis, particularly in patients with recent gastrointestinal injury.

This paper was previously presented as a QuickShot at the Southeastern Surgical Congress Annual Meeting in New Orleans in February 2025.

## Case presentation

**Figure 1:**
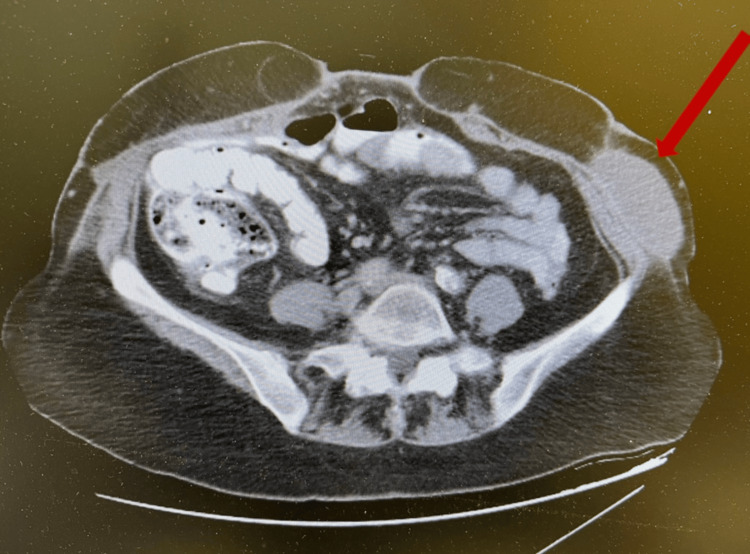
CT scan showing abdominal wall abscess and necrosis Abdominal wall abscess (red arrow) CT: computed tomography

General Surgery was consulted for a 77-year-old female following a total abdominal hysterectomy with bilateral salpingo-oophorectomy. She presented with diffuse, intractable abdominal pain and persistent vomiting. Clinical examination revealed peritonitis and greenish-brown drainage from her Pfannenstiel incision, accompanied by diffuse abdominal pain, guarding, and abdominal rigidity. Upon removal of the wound dressing, meconium-like, greenish-brown fluid was noted saturating the bandage, raising suspicion of bowel perforation and bilious drainage. A CT scan was obtained (Figure 1), and emergent exploratory laparotomy was performed, revealing a small bowel injury.

**Figure 2:**
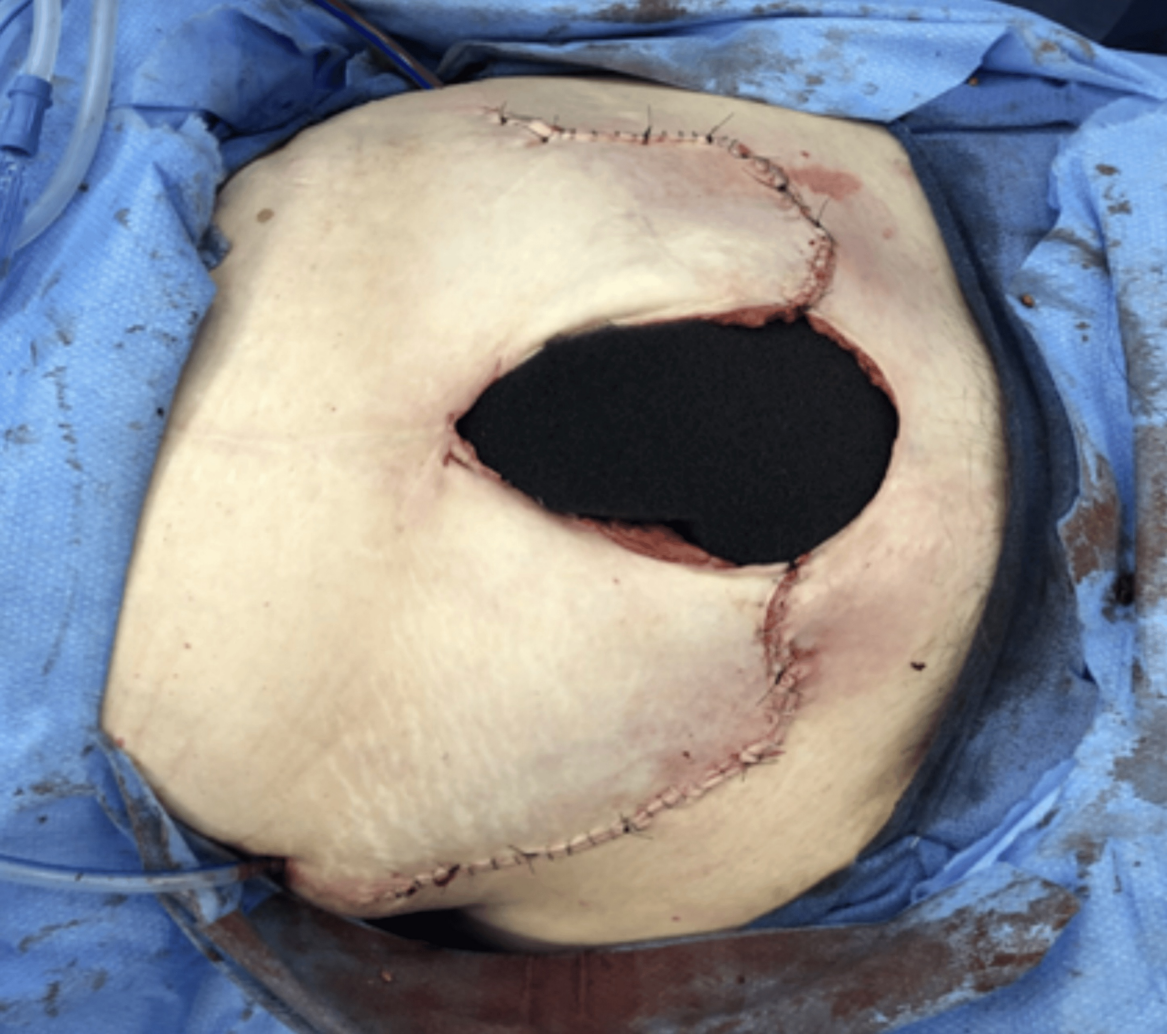
Open abdominal wall defect managed with AbThera

Segmental small bowel resection was done, and pathology of the specimen confirmed a central perforation with extensive inflammation, hemorrhage, and granulation tissue. The patient had extensive necrotizing myonecrosis, necessitating debridement of the anterior abdominal wall musculature. The abdomen was left open, and an AbThera vacuum-assisted closure system (manufactured by KCI) was placed (Figure 2).

**Figure 3:**
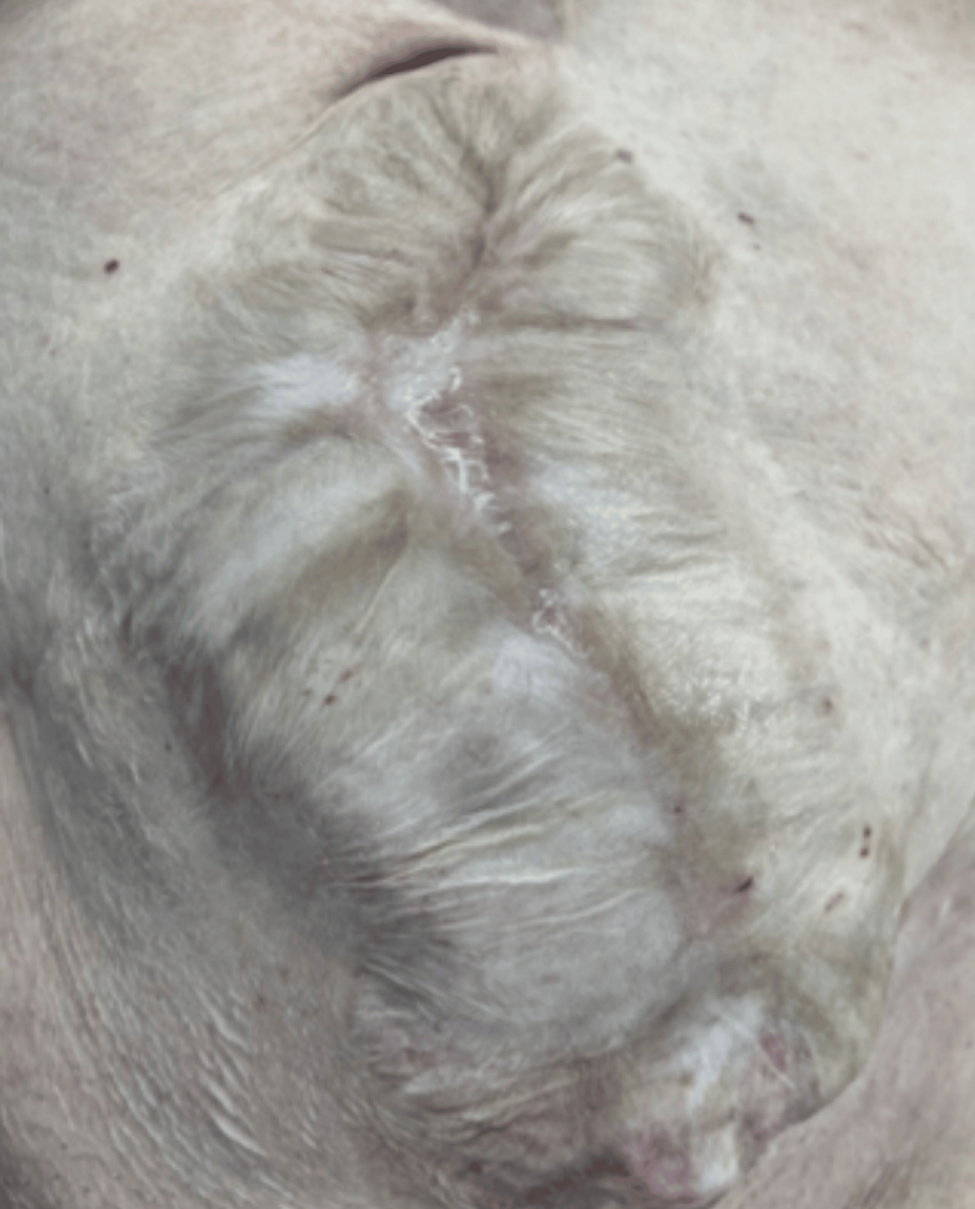
Giant ventral hernia

**Figure 4:**
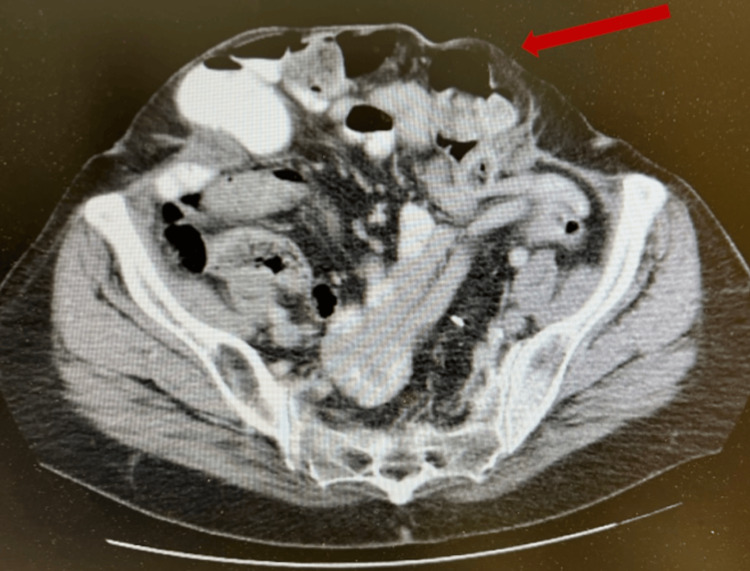
CT scan demonstrating a giant ventral hernia with significant loss of domain, characterized by protrusion of abdominal contents beyond the confines of the abdominal cavity Giant ventral hernia (red arrow) CT: computed tomography

The following day, the patient underwent re-exploration and anastomosis of the small bowel. Over the next two weeks, she required multiple surgical debridements to remove necrotic skeletal muscle and subcutaneous tissue from the abdominal wall. The overlying skin was closed, except at the site of the ventral hernia defect, which was left open for a planned future repair (Figures 3, 4).

Empiric/broad-spectrum antibiotic therapy with IV piperacillin-tazobactam was initiated, targeting a presumed polymicrobial bacterial infection. Intraoperative cultures from subcutaneous tissue and an abdominal abscess revealed *Candida albicans* as the sole pathogen. Gram staining, aerobic, and anaerobic cultures exhibited no growth. Infectious disease was consulted, and antifungal therapy with fluconazole was initiated. The patient demonstrated progressive clinical improvement and was discharged with a plan to address the ventral hernia on an elective basis. 

**Figure 5:**
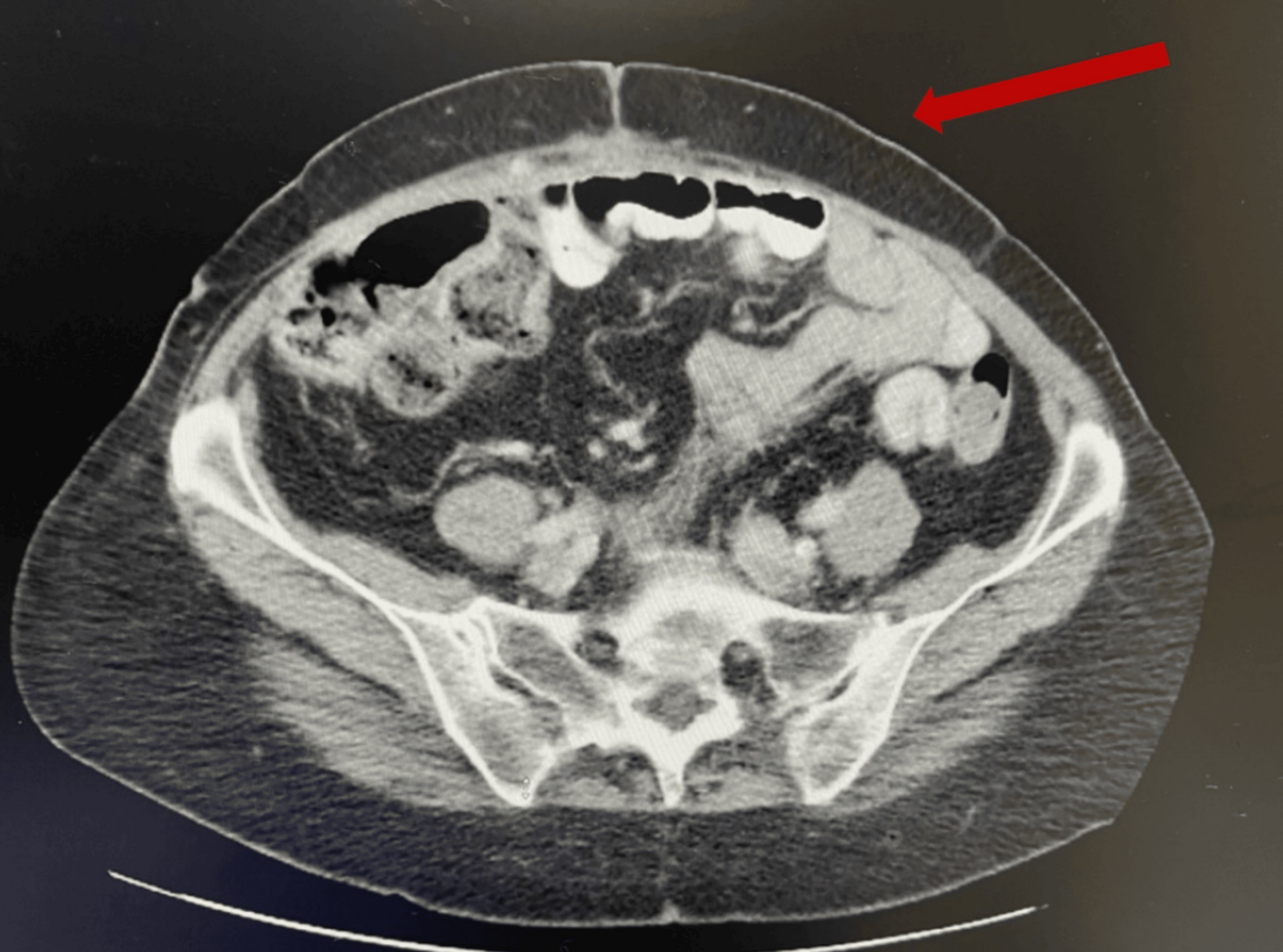
CT scan following retro-rectus component separation, transverse abdominis release, and mesh placement for reinforcement to repair the giant ventral hernia Repaired ventral hernia (red arrow) CT: computed tomography

She later underwent complex ventral hernia repair, which included retro-rectus component separation, transverse abdominis release, and mesh placement for reinforcement [6]. The patient has continued to do well at follow-up (Figure 5).

## Discussion

This case highlights a rare, life-threatening occurrence of *Candida albicans* necrotizing myonecrosis following a small bowel perforation in a relatively healthy elderly patient. Fungal infections of this severity are typically associated with immunosuppression; however, our case underscores the need to consider fungal pathogens, such as *Candida*, in the differential diagnosis of postoperative infections following gastrointestinal surgeries. While the majority of necrotizing soft tissue infections are caused by bacterial species, fungal infections remain exceedingly rare and present unique challenges in both diagnosis and management. This case serves as a reminder of the importance of maintaining a broad differential, particularly in patients with unexpected postoperative complications, to ensure timely and effective treatment. 

Common triggers for necrotizing fasciitis include surgical procedures and penetrating injuries, with most cases occurring in immunocompromised individuals or those with predisposing factors such as chronic disease, diabetes, IV drug use, advanced age, burns, malnutrition, malignancy, trauma, or renal failure [7]. Necrotizing soft tissue infections (NSTIs) affect about 1,000 individuals annually in the United States and are classified based on microbial etiology, infection depth, or anatomical location [2]. Type I NSTIs, the most common subtype, are polymicrobial and typically involve a synergistic mix of gram-positive cocci, gram-negative bacilli, and anaerobes. Type II NSTIs are monomicrobial, usually caused by *Streptococcus pyogenes*, either alone or with *Staphylococcus aureus*, and are associated with toxic shock syndrome, with prevalence rising alongside MRSA. Type III NSTIs, characterized by mortality rates of 30-40%, are monomicrobial and predominantly caused by Vibrio species. These infections progress rapidly and require prompt intervention. Type IV NSTIs, the rarest subtype, are fungal in origin, primarily involving Candida species and Zygomycetes in immunocompetent individuals. With mortality rates exceeding 47%, they are most common in patients with traumatic burns or wounds and require immediate treatment due to their rapid progression [8]. 

*Candida albicans*, typically a commensal organism of the gastrointestinal tract, can cause fulminant infection under rare circumstances. Though *Candida* is a rare culprit in necrotizing myonecrosis, this case illustrates the organism's opportunistic potential. In contrast, bacterial pathogens remain the predominant cause of necrotizing infections. In a study by Sudarsky et al., *β-hemolytic Streptococcus* was the most frequently cultured organism, followed by *Staphylococcus aureus*, *alpha-hemolytic* *Streptococcus*, and *Escherichia coli* [5]. In a retrospective study of 182 patients with necrotizing fasciitis, 70 had cultures positive for *β-hemolytic Streptococcus*, 58 for *Escherichia coli*, 27 for *Staphylococcus aureus*, and 7 had cultures positive for *Candida* species [9]. These findings support the inclusion of broad-spectrum antibacterial agents in initial empiric therapy, but they also underscore the importance of culture-guided treatment, especially in cases unresponsive to standard therapy, where uncommon fungal pathogens may be involved.

Fungal etiologies of necrotizing myonecrosis should be considered in severe infections following abdominal trauma. Eisen and Brown reported a case of Fournier’s gangrene in a 50-year-old male after abdominal trauma from a motor vehicle accident, with *Candida albicans* and *Candida tropicalis* confirmed via histopathology [10]. Atallah et al. described *Candida albicans* necrotizing fasciitis following an elective lumbar spine procedure in an immunocompetent patient without risk factors [11]. Although rare, there is a growing body of literature reporting *Candida* as a causative organism in necrotizing tissue infections. While surgical debridement remains the cornerstone for management to reduce mortality in these cases, prompt culture and pathogen identification are essential for guiding targeted management.

## Conclusions

This case underscores the critical need for early diagnosis and aggressive surgical debridement of necrotizing myonecrosis. Prompt exploration, definitive surgical debridement, and obtaining tissue cultures are of great importance when a patient presents with signs of a necrotizing infection in the abdominal wall. While bacterial pathogens are most commonly identified in such cases, the potential for fungal causes, such as *Candida*, should not be overlooked in patients presenting with necrotizing infection.
